# Cowden Syndrome With a Co-existing Lynch Syndrome Risk Mutation

**DOI:** 10.7759/cureus.97073

**Published:** 2025-11-17

**Authors:** Ahmed Alajaimi, Yaser Alderazi, Safa Alshaikh

**Affiliations:** 1 Department of General Surgery, Salmaniya Medical Complex, Manama, BHR; 2 Department of Pathology, Salmaniya Medical Complex, Manama, BHR

**Keywords:** adenomatoid nodules, : cowden syndrome, lynch syndrome, pediatric thyroid nodule, pms2, pten

## Abstract

Thyroid nodules in children are rare but clinically significant due to their relatively high malignancy risk compared with adults. Genetic predisposition syndromes, such as PTEN hamartoma tumor syndrome (PHTS; Cowden syndrome) and Lynch syndrome, further complicate presentation, surveillance, and management. We present a 15-year-old female with a thyroid nodule, ultimately found to harbor dual germline mutations in the PTEN and PMS2 genes. This unusual co-occurrence highlights the diagnostic and management challenges associated with overlapping hereditary cancer syndromes. Comprehensive clinical assessment, imaging, histopathology, and genetic testing established the diagnosis. The case emphasizes the importance of multidisciplinary surveillance and early genetic counseling in patients presenting with thyroid nodules and strong familial cancer histories.

## Introduction

Pediatric thyroid nodules are uncommon, with an estimated prevalence of 1-1.5%, yet their likelihood of malignancy can reach up to 25%, markedly higher than in adult populations. While many nodules are sporadic, inherited cancer predisposition syndromes can significantly influence risk, prognosis, and surveillance strategies.

PTEN hamartoma tumor syndrome (PHTS), most notably manifested as Cowden syndrome, arises from germline PTEN mutations and is characterized by hamartomas and increased lifetime risks of thyroid, breast, renal, and endometrial cancers [1,2,3]. Pediatric patients with PHTS often present early with thyroid nodular disease, which may precede malignant transformation [4,5,6].

Lynch syndrome, caused by germline defects in mismatch repair (MMR) genes (e.g., MLH1, MSH2, MSH6, PMS2), significantly raises the risk of colorectal, endometrial, and various other tumors [7-9]. Among these, PMS2 mutations typically have lower penetrance than MLH1 or MSH2 mutations, but carriers still face substantial cancer risk.

The co-occurrence of PTEN and PMS2 germline mutations in a single individual is exceedingly rare, especially in pediatric patients [8,9]. We report a case of a 15-year-old female with thyroid nodules whose evaluation revealed pathogenic variants in both PTEN and PMS2, and we explore the clinical implications of this overlap.

## Case presentation

A 15-year-old female was referred for evaluation of a gradually enlarging anterior neck swelling of one year’s duration. She denied dysphagia, dyspnea, hoarseness, thyroid-related symptoms (e.g., heat intolerance, tremors), or systemic complaints. Her past medical history was unremarkable, and no prior radiation exposure was reported. The family history was notable for maternal relatives with colorectal and endometrial cancers.

On physical examination, she was euthyroid and in stable condition. Inspection of the neck revealed a right-dominant anterior midline thyroid swelling that moved with swallowing and was non-tender. Palpation identified a firm, smooth nodule in the right thyroid lobe. There was no palpable cervical lymphadenopathy, and Pemberton’s sign was negative. No signs of thyroid dysfunction (e.g., tremor, lid lag) were observed.

**Figure 1:**
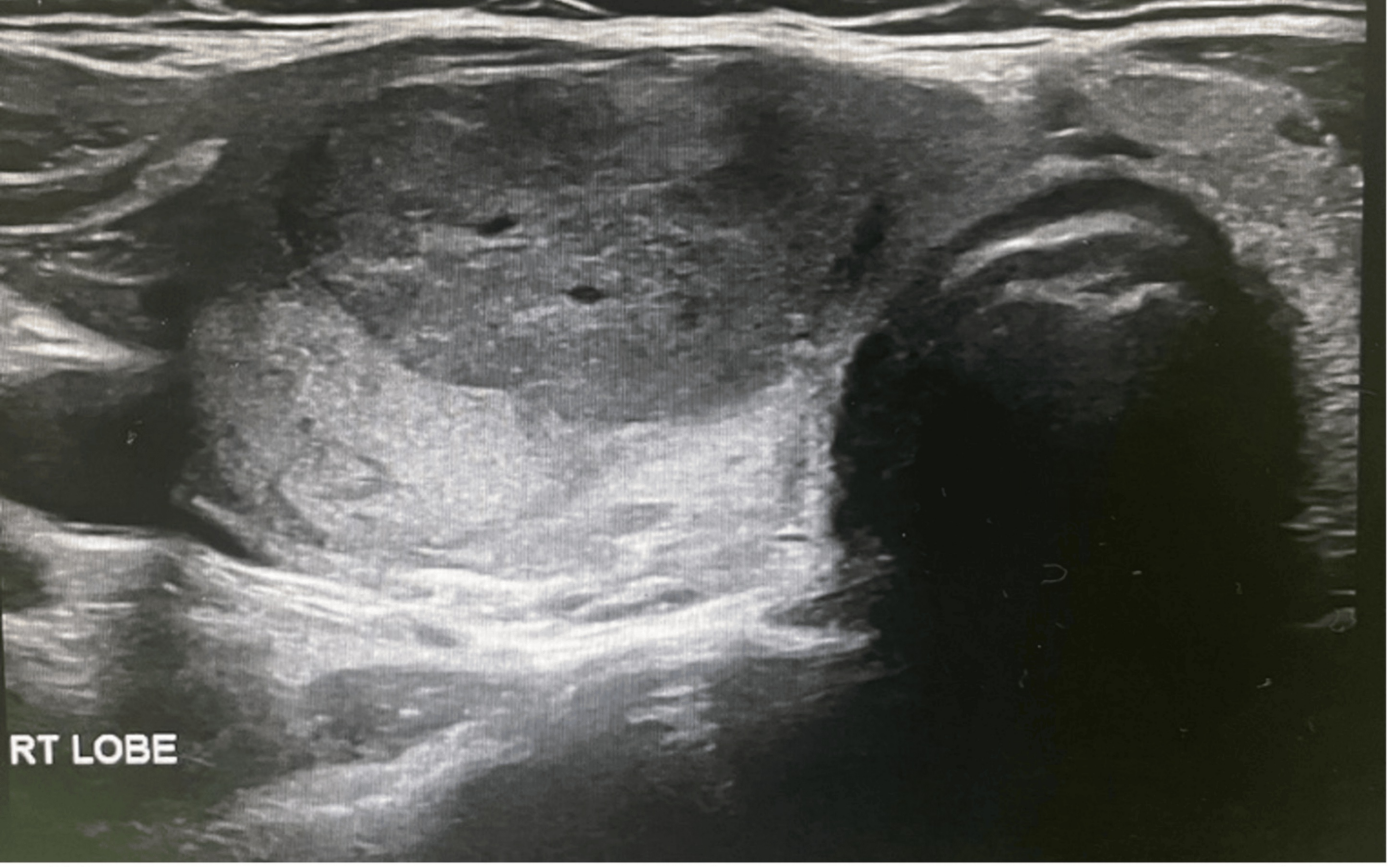
The right lobe of the patient’s thyroid gland.

**Figure 2:**
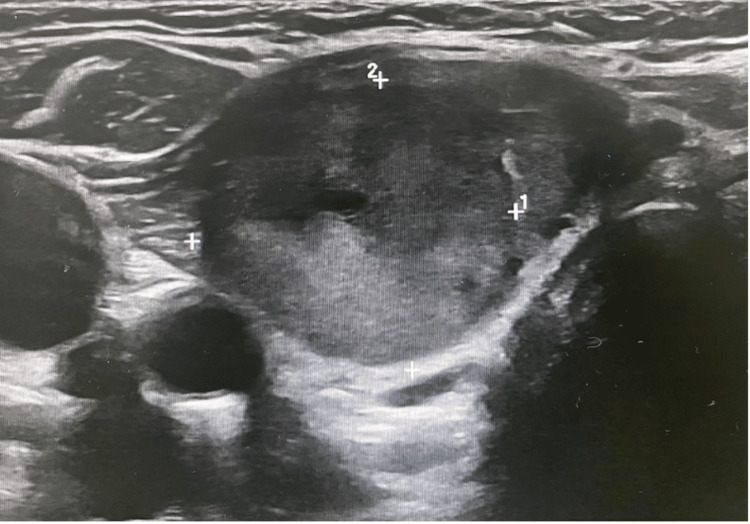
The left lobe of the patient’s thyroid gland.

Neck ultrasound demonstrated enlargement of the right thyroid lobe (2.0 × 2.6 × 5.1 cm) containing multiple isoechoic nodules, the largest measuring 1.3 × 2.3 × 2.0 cm. These nodules showed peripheral vascularity without cystic degeneration or calcification. The left lobe measured 1.2 × 1.3 × 3.2 cm and contained only tiny nodules (<2 mm). Bilateral prominent cervical lymph nodes were also noted (largest approximately 3.2 × 1 cm). The overall impression was TIRADS 3 (Figures 1-2).

Two fine-needle aspiration (FNA) biopsies were performed, both yielding atypia of undetermined significance (AUS)/follicular lesion of undetermined significance (FLUS) [3,10]. Given the indeterminate cytology and interval growth, the patient underwent an elective right hemithyroidectomy. Intraoperatively, the right lobe contained a well-circumscribed nodule with no evidence of extracapsular extension or suspicious lymphadenopathy. The recurrent laryngeal nerve and ipsilateral parathyroid glands were visualized and preserved.

**Figure 3:**
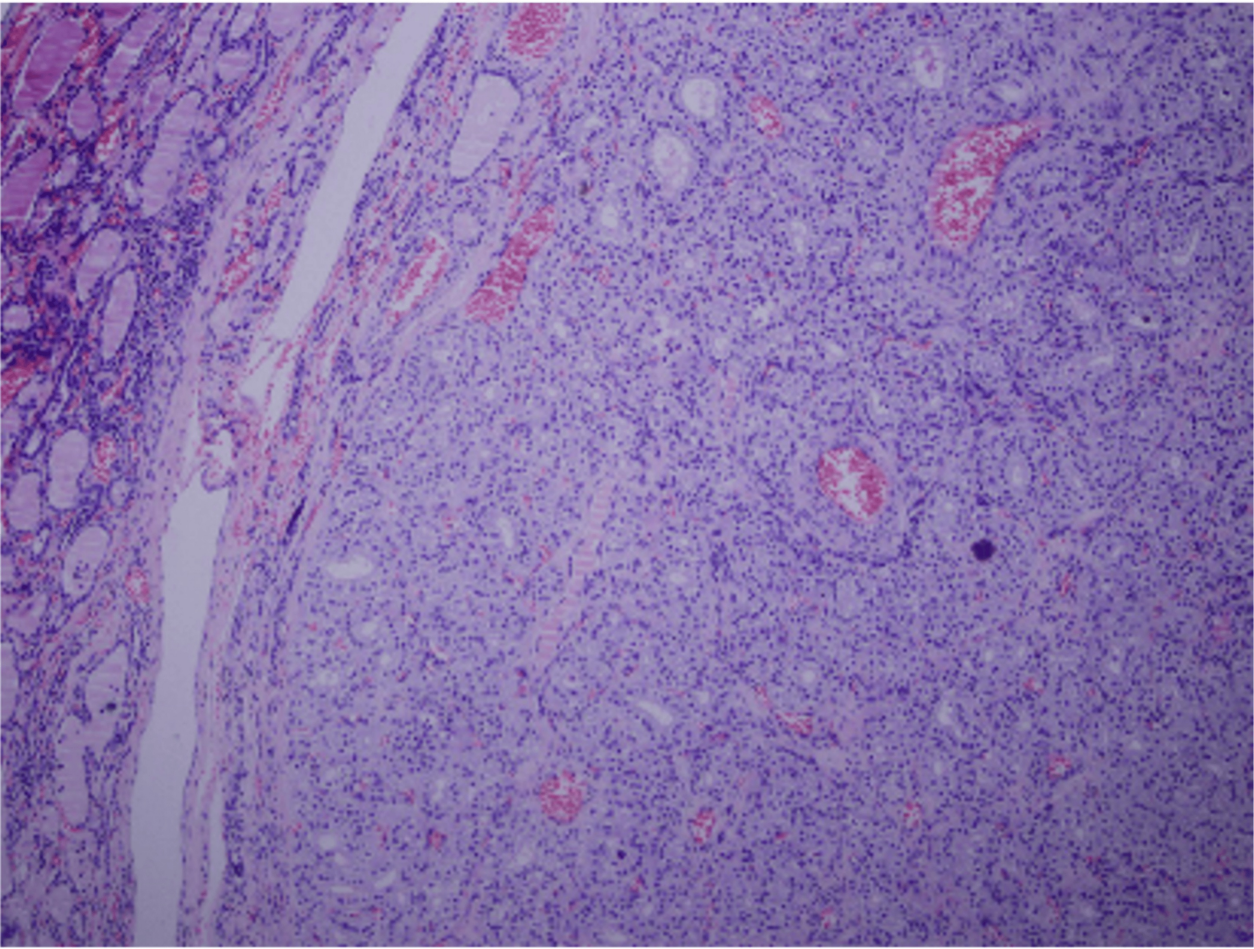
Histopathological features of the lesion.

**Figure 4:**
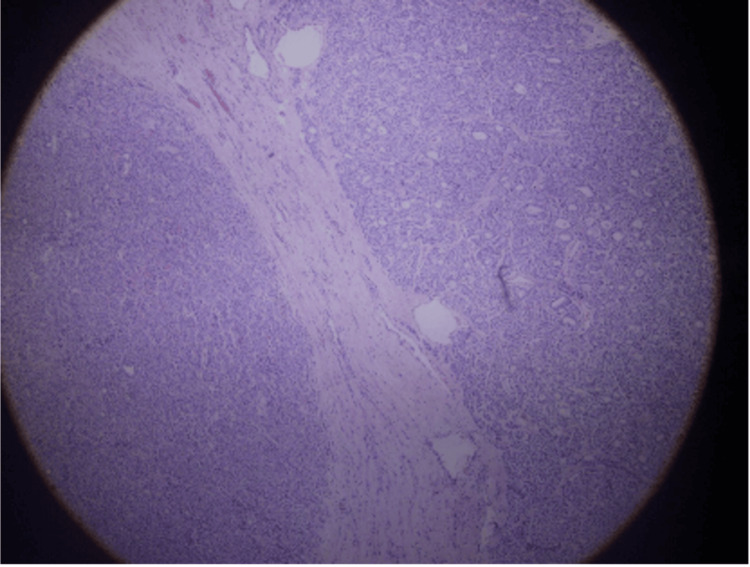
Histopathological slide of the lesion.

Microscopic examination revealed multiple encapsulated adenomatoid nodules composed of compressed follicles lined by follicular cells with eosinophilic cytoplasm and minimal nuclear atypia. Focal lymphocytic infiltration associated with fibrosis was present. Incidental benign thymic and parathyroid tissue were also identified. No malignancy was detected (Figures 3-4).

The abnormal thyroid morphology, along with the histological findings, indicated the need for genetic analysis. Testing revealed a pathogenic truncating PTEN mutation, c.388C>T, p.(Arg130*), confirming Cowden syndrome, as well as a pathogenic PMS2 frameshift variant, c.2192_2196del, p.(Leu731Cysfs*3), consistent with an increased risk for Lynch syndrome (hereditary non-polyposis colorectal cancer type 4).

## Discussion

This case illustrates the rare convergence of PTEN and PMS2 germline mutations in a pediatric patient presenting with thyroid nodules. In children with PHTS, benign nodular thyroid disease is common, and differentiated thyroid carcinoma (DTC) can occur [3,4,5,11]. Recent data suggest that the incidence of DTC in childhood PHTS ranges from 4 to 12% across cohorts, supporting the need for early surveillance [11]. Jonker LA et al. recommend initiating thyroid ultrasound screening from age 10 onward in PHTS carriers [5].

Bormans EM et al. (2025) reported that 84% of pediatric PHTS patients develop thyroid abnormalities and that 16% undergo (hemi)thyroidectomy due to nodule growth. Similarly, Plamper M et al. found that thyroid abnormalities may present as early as 4-5 years of age [4]. These findings reinforce the importance of proactive ultrasound surveillance and multidisciplinary management in PHTS patients.

The coexistence of a PMS2 pathogenic variant introduces additional surveillance complexity. Although PMS2 mutations have comparatively lower penetrance, carriers remain at increased lifetime risk for colorectal and endometrial cancers [7-9]. Accordingly, the management of patients with dual mutations should integrate both PTEN-related and Lynch syndrome-related surveillance protocols [3,6].

This case underscores the importance of early genetic evaluation in pediatric thyroid nodular disease, even in the absence of a family history of thyroid cancer [1,4,5]. In patients with multifocal nodules or histologic features such as multiple adenomatoid nodules, clinicians should maintain a low threshold for germline testing.

## Conclusions

This case demonstrates that pediatric thyroid nodular disease may herald underlying hereditary cancer syndromes. The rare concurrence of PTEN and PMS2 pathogenic variants necessitates comprehensive, multidisciplinary care. Early genetic diagnosis, tailored surveillance, and family counseling are essential to optimize outcomes and mitigate cancer risk.
